# Postoperative opioid use in Norway—a population-based observational study on patterns of long-term use

**DOI:** 10.1186/s40360-024-00805-y

**Published:** 2024-10-25

**Authors:** Sara Magelssen Vambheim, Vidar Hjellvik, Ingvild Odsbu, Svetlana Skurtveit, Christopher Ekholdt, Lars Petter Granan, Audun Stubhaug, Per-Jostein Samuelsen

**Affiliations:** 1 Department of Research and Development, Division of Emergencies and Critical Care, Oslo University Hospital, Oslo, Norway; 2 Department of Pain Management and Research, Oslo University Hospital, Oslo, Norway; 3 Institute of Clinical Medicine, University of Oslo, Oslo, Norway; 4 Department of Chronic Diseases, Norwegian Institute of Public Health, Oslo, Norway; 5 Regional Medicines Information and Pharmacovigilance Centre (RELIS), University Hospital of North Norway, Tromsø, Norway

**Keywords:** Opioids, Opioid analgesics, Surgery, Long-term use, Post-operative opioid use

## Abstract

**Background:**

The utilization patterns of opioid analgesics and the proportion of long-term opioid use after surgery in Norway is largely unknown.

**Methods:**

This study aimed to estimate the proportion of one-year long-term prescription opioid use among all Norwegian postoperative opioid users. Complete data from central health registries (NPR, NorPD, Statistics Norway, CoDR) were linked via the personal identification number unique to all citizens. The study period was January 1st 2010 until December 31st 2019. Long-term opioid use was defined as at least two opioid dispensings within two subsequent 90-day periods, with a minimum average use of 10 MME/day for the first 90 days.

**Results:**

The study population consisted of 693 495 post-operative opioid users (53.6% women), whereof 73.2% had not used opioids the year before surgery (new users). Among the postoperative opioid users, 3.8% were one-year long-term opioid users. The corresponding figures for new and previous opioid users were 0.4% and 13.1%, respectively. The highest proportions of long-term opioid use were found after transluminal endoscopy, eye surgery and assessments related to surgical procedures. In previous opioid users, the proportion of one-year long-term use was higher among women than men in all age groups, a difference that increased with age.

**Conclusions:**

The proportion of postoperative long-term opioid use in Norway is generally low. We detected higher proportions of long-term opioid use after certain types of surgery, but our crude surgery definition warrants further examination. Previous opioid users pose a particular challenge in the management of postoperative pain.

**Trial registration:**

The study used national health registry data from the period 2010–2019. A pre-registered analysis plan is available at Open Science Framework.

**Supplementary Information:**

The online version contains supplementary material available at 10.1186/s40360-024-00805-y.

## Introduction

The overall prevalence of prescription opioid use in Norway is stable, but there have been some changes in types of opioids prescribed [1]. Although the prevalence is low compared to several other countries, Norway had the highest one-year prevalence in opioid use among the Scandinavian countries between 2006 and 2017, due to the frequent use of paracetamol/codeine for short-term moderate pain. Over the last decade, there has been an increase in the use of oxycodone and tramadol, and a decrease in the use of codeine in Norway [1]. No national guidelines for postoperative pain management exist in Norway, and internal guidelines for opioid use in different hospitals and surgery departments vary. Thus, little is known about prescription practices and use of opioids after surgery in Norway.

A few Norwegian registry-based studies have focused on certain types of surgical procedures [2, 3], e.g. lumbar spine surgery and total hip arthroplasty. This has led to increased knowledge about prevalence of long-term opioid use and opioid prescription practice after selected surgical procedures. International studies show that both minor and major surgery are risk factors for long-term opioid use, a risk that is higher compared to other medical procedures [4, 5]. However, an overall view of postoperative opioid use in general is lacking.

Management of postoperative pain is two-fold. At first the goal is to minimize perioperative acute pain and promote mobilization for the first postoperative period. Secondly, the management of pain and risks associated with opioid use in the following weeks or months must be carefully balanced. Most patients that receive opioid prescriptions at discharge from hospitals are postoperative patients [6]. Moreover, overprescribing of opioids increases the risk of pharmaceutical opioid overdose [7], as well as long-term use and postoperative complications such as infections and gastrointestinal complications [8]. However, interventions that include standardized guidelines for pain management after surgery, followed by a focus on risks of opioid use, have proven successful in reducing the amount of opioids prescribed and used postoperatively [9]. It has also been found that restrictive prescription guidelines reduce the risk of long-term opioid use and total opioid consumption, without increased pain intensity or reduced therapeutic satisfaction [10, 11].

This study examined the proportion of one-year long-term postoperative opioid use in the Norwegian population that filled a postoperative opioid prescription. The main aim was to estimate the proportion of one-year long-term (1yrLT) postoperative opioid users. Secondly, we examined the same proportion in subgroups, such as surgery type, new versus previous opioid use, age, sex, use of benzodiazepines, and geographical region.

## Methods

### Data sources and study period

In this registry-based study, complete national registry data were linked (utilizing pseudonyms based on the personal identification numbers) in the period January 1, 2010 to December 31, 2019 from the Norwegian Patient Registry (NPR), the Norwegian Prescription Database (NorPD), the Cancer Registry of Norway (CRN), the Norwegian Population Registry, Statistics Norway and the Norwegian Cause of Death Registry (CoDR).

The NPR provides data from the somatic hospitals, specialist health care institutions, and outpatient clinics, e.g., demography (sex, age), treatment location, codes for surgical procedures [12] and date of assessment, treatment or discharge. Surgical procedures and diagnoses were defined based on The Nordic Medico-Statistical Committee (NOMESCO) Classification of Surgical Procedures (NCSP) and International Statistical Classification of Diseases and Related Health Problems (ICD-10) codes (The Directorate of eHealth, 2023). We included all NCSP main chapters, with further subdivision down to the third digit (Additional file 1). For simplicity, we refer to chapter F, G and K as “Heart”, “Chest” and “Urinary organs”, respectively.

The NorPD contains data on all dispensed drugs from Norwegian pharmacies [13]. Drugs are classified according to the World Health Organization’s Anatomical Therapeutic Chemical (ATC) classification system [14]. The CRN provides data on incident malignancies (ICD-10 codes) [15]. Data on emigration was retrieved from Statistics Norway and information on date of death was extracted from the CoDR.

### Study population

**Fig. 1:**
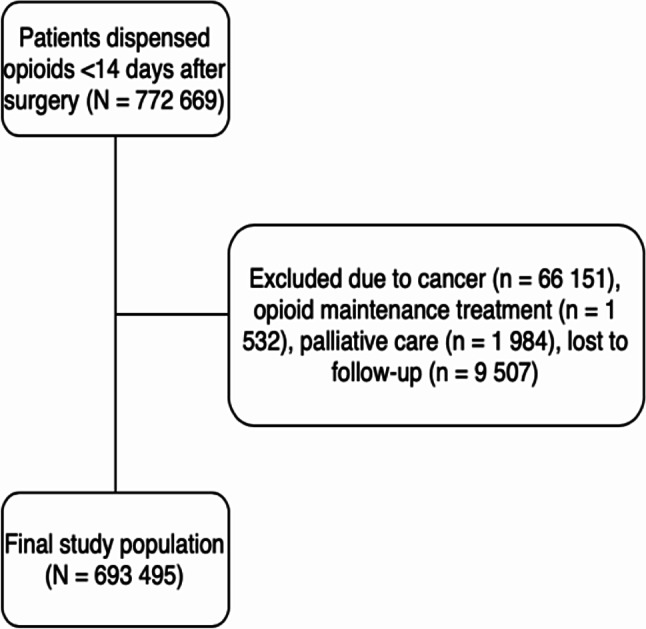
Flowchart of patient inclusion

We included all patients aged ≥15 years that had at least one opioid dispensing between January 1, 2011 and December 31, 2018, and a surgery within 14 days prior to this dispensing (Fig. 1). The index date was set to the date of the first registered postoperative opioid dispensing (index dispensing). We set the date of the surgery to the date of the registered procedure within a hospital stay, or otherwise to the hosptal admission date.

### Exclusion criteria

Patients with a registered cancer diagnosis, drug reimbursement code for palliative care or receiving opioid maintenance treatment within the 365 days prior to the index date, or with less than 365 days follow-up data after the index date were excluded. Cancer was defined as codes belonging to chapter C of the ICD-10, from CRN and NPR, respectively, and/or drug reimbursement codes for palliative care (reimbursement code -90). Opioid maintenance therapy was defined as dispensing of drugs within the ATC group N07BC [14]. The minority of patients receiving drugs within N07BC for pain, e.g., methadone tablets, was therefore excluded. A visual presentation of the study setup is available in the supplementary (Additional file 2).

### Opioid analgesics and definition of long-term use

Opioid analgesics were defined as all drugs classified in ATC group N02A. We adapted the methods by Hamina and colleagues [16] for the definition of long-term opioid use. This definition requires at least two opioid dispensings within two subsequent 90-day periods, i.e., the dispensings had to be separated by 91–180 days, and that ≥90 administration units are dispensed during the first period. We opted to use morphine milligram equivalents (MME) instead, as in the additional analyses by Hamina et al. We required at least 900 MMEs being dispensed within the first 90 days from (and including) the first of these dispensings, i.e., minimum average use of 10 MME/day for the first 90 days. The first of these dispensings defined the start of a long-term use episode. Dispensings before index date were ignored in the computation of long-term use episodes. MME was calculated as previously described [16] and included both tablets, capsules, transdermal patches and mixtures.

A long-term use episode ended if the gap between two dispensings was >180 days, at the date of death, emigration, or end of the study period. A new long-term treatment episode started if the criteria again was met. Thus, one person could have several long-term use episodes over the study period. The duration of the long-term episode was defined as the number of days between the first and last dispensing, plus the estimated duration of the last dispensing (derived from the average MMEs per day; limited to a maximum of 180 days), in accordance with Samuelsen and colleagues [17].

**Fig. 2:**
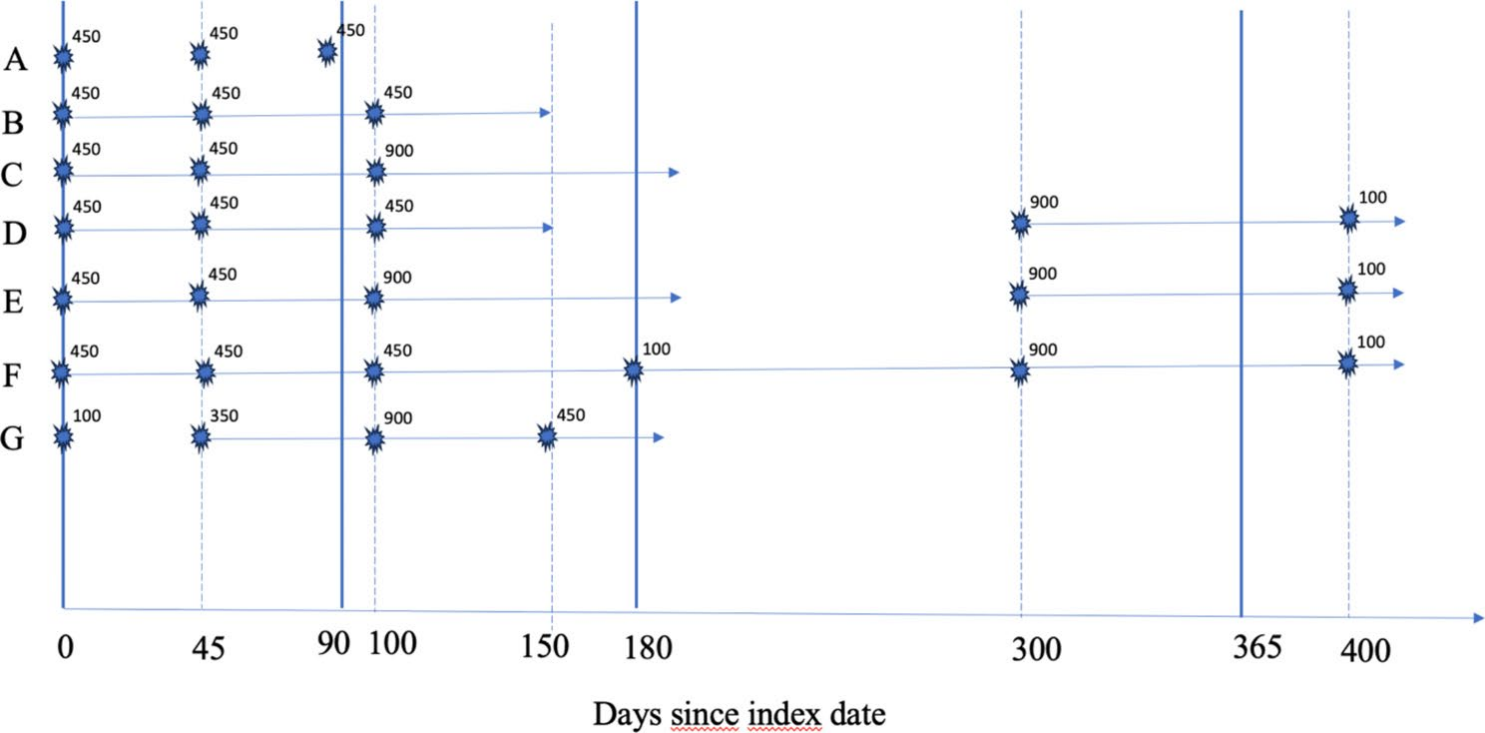
Computation of one-year long-term opioid use in 6 hypothetical patients. Patient A have no dispensings in consecutive 90-day-periods and is not a long-term user. Patients B–F have dispensings in two consecutive 90-day-periods, and ≥900 MMEs in the first 90 days, and are thus long-term users. The long-term episodes stops when the gap to the next dispensing is >180 days, and the duration of the last dispensing within that episode is added. Patients D and E have a second long-term treatment episode starting when the criteria are once again met. Patient G only fulfills the criteria from the second dispensing and onwards. Patient E and F are one-year long-term (1yrLT) opioid users, since day 90, 180, and 365 are all covered by long-term treatment episodes

For this study, we focus on one-year long-term (1yrLT) opioid users, defined as having day 90, 180 and 365 after index day covered by long-term treatment episodes with opioids. Figure 2 illustrates the computation of long-term periods and 1yrLT use for 6 hypothetical patients (Fig. 2).

### Statistical analysis

All analysis were conducted using R (R Foundation for Statistical Computing, Vienna, Austria. https://www.R-project.org/). We calculated the proportion (%) of postoperative opioid users that developed 1yrLT use, stratified on previous use (at least one opioid prescription 1-365 days before index date) yes/no, surgery type (NCSP main chapters), age groups (15–39, 40–59, 60–79, 80+), type of opioid at first postoperative prescription (codeine, tramadol, oxycodone, morphine, other), use of benzodiazepine/Z-drugs (zopiclone, or zolpidem) the year before index date, sex, and health region (Northern, Central, Western, South-Eastern). As the study population is large (~700 000), the confidence intervals for the proportions are small, and therefore in most cases omitted.

An individual was considered a previous opioid user if at least one opioid prescription had been filled the year before the index date. Users with no opioid prescription filled in the same time window were considered as new users of opioids. Opioid type refers to the first postoperative opioid prescription.

Healthcare region reflects where the surgical procedure was performed, and is divided into the four health regions in Norway (Northern-, Central-, Western- or South-Eastern Regional Health Authority). Benzodiazepines or benzodiazepine-related drugs included the ATC codes N03AE01 (clonazepam), N05BA (benzodiazepines-anxiolytics), N05CD (benzodiazepines-hypnotics), and N05CF (benzodiazepine-related drugs; the term Z-drugs is used in the text).

## Results

The study population consisted of 693 495 surgery patients that filled an opioid prescription within 14 days after the surgery (53.6% women, mean age 49.2 years) (Table 1). Women were slightly older (50.7 years) than men (47.7 years). In total 73.2% were new users of opioids (women 70.1%; men 76.8%). Previous users of opioids were older (54.3 years) and more likely women (59.8%), compared to new users (47.3 years; women 51.3%). Surgery within the locomotor system constituted the highest proportion of surgical procedures, followed by surgery within the digestive organs and spleen, and minor surgical procedures (Table 1). A higher proportion of women (24.6%) than men (13.5%) received benzodiazepines and Z-drugs the year prior to surgery.

**Table 1:** Characteristics of the study population

	Men *N* = 321 490	Women *N* = 372 005	Total *N* = 693 495
*Age*,* mean (SD)*	47.7 (18.1)	50.7 (19.2)	49.2 (18.8)
*Age groups*,* n (%)*			
15–39	114 193 (35.5)	112 939 (30.3)	227 132 (32.7)
40–59	115 899 (36.0)	127 795 (34.3)	243 694 (35.1)
60–79	80 804 (25.1)	106 411 (28.6)	187 215 (27.0)
80+	10 594 (3.3)	24 860 (6.7)	35 454 (5.1)
*Surgery*,* n (%)*			
Nervous system	17 517 (5.4)	20 445 (5.5)	37 962 (5.4)
Endocrine organs	200 (0.1)	645 (0.1)	845 (0.1)
Eye, eye region	5070 (1.6)	7070 (1.9)	12 140 (1.7)
Ear, nose, sinus and larynx	16 671 (5.2)	10 983 (2.9)	27 654 (4)
Teeth, jaw, mouth, pharynx	17 440 (5.4)	23 926 (6.4)	41 366 (5.9)
Heart	4051 (1.2)	1701 (0.4)	5752 (0.8)
Chest	2761 (0.8)	1183 (0.3)	3944 (0.5)
Mammae	563 (0.1)	5196 (1.4)	5759 (0.8)
Digestive organs, spleen	37 484 (11.6)	40 251 (10.8)	77 735 (11.2)
Urinary organs	8453 (2.6)	3263 (0.8)	11 716 (1.7)
Female genitalia	23 (0.0)	20 903 (5.6)	20 926 (3.0)
Birth, pregnancy	N/A	2097 (0.5)	2097 (0.3)
Locomotor system	142 600 (44.3)	157 038 (42.2)	299 638 (43.2)
Peripheral veins, lymph system	4160 (1.3)	5577 (1.5)	9737 (1.4)
Skin	24 110 (7.5)	18 153 (4.9)	42 263 (6.1)
Minor surgical procedures	28 396 (8.8)	33 046 (8.9)	61 442 (8.8)
Transluminal endoscopy	10 576 (3.2)	15 178 (4.1)	25 754 (3.7)
Assessments related to surgical procedures	1342 (0.4)	5288 (1.4)	6630 (0.9)
Organ or tissue extraction for transplantation	72 (0.0)	62 (0.0)	134 (0.0)
*Health region*,* n (%)*			
Northern	30 491 (9.4)	34 239 (9.2)	64 370 (9.3)
Central	46 477 (14.4)	54 022 (14.5)	100 499 (14.5)
Western	63 098 (19.6)	70 520 (18.9)	133 618 (19.2)
South-Eastern	180 806 (56.2)	213 077 (57.3)	398 883 (57.5)
*Previous opioid use*,* n (%)*	74 704 (23.2)	111 264 (29.9)	185 968 (26.8)
*New opioid use*,* n (%)*	246 786 (76.8)	260 741 (70.1)	507 527 (73.2)
*Benzodiazepine and Z-drugs, n (%)*			
Benzodiazepines or Z-drugs	43 521 (13.5)	91 530 (24.6)	135 051 (19.5)
Benzodiazepines alone	14 646 (4.5)	26 705 (7.1)	41 351 (5.9)
Z-drugs	20 854 (6.4)	43 793 (11.7)	64 647 (9.3)
Both benzodiazepines and Z-drugs	8021 (2.4)	21 032 (5.6)	29 053 (4.1)

*Note* Surgery is classified according to the NOMESCO classification system (NCSP codes)

*SD* standard deviation, *N/A* not applicable

### First dispensed opioids after surgery

The most frequently dispensed opioids after surgery were codeine in combination with paracetamol (58.3%), tramadol (38.6%) and oxycodone (3.8%) (Table 2). Previous opioid users were more frequently dispensed oxycodone or morphine compared to new users. In total 35.0% had their first postoperative dispensing at the day of surgery, 19.6% the day after surgery, and 45.4% between day 2 to 13 after surgery.

**Table 2:** Distribution of opioid type at index dispensing in the total study population and among one-year long-term users in previous and new opioid users

All opioid users			
	Previous opioid users *N* = 185 968	New opioid users *N* = 507 527	Total *N* = 693 495
*Opioid type*,* n (%)*			
Codeine	101 649 (54.7)	302 411 (59.6)	404 060 (58.3)
Tramadol	71 843 (38.6)	195 962 (38.6)	267 805 (38.6)
Oxycodone	11 190 (6.0)	14 828 (2.9)	26 018 (3.8)
Morphine	741 (0.4)	300 (0.1)	1041 (0.2)
Other	6094 (3.3)	2125 (0.4)	8219 (1.2)

1yrLT users			
	*N* = 24 382	*N* = 1939	*N* = 26 321
*Opioid type*,* n (%)*			
Codeine	7430 (30.5)	493 (25.4)	7923 (30.1)
Tramadol	10 893 (44.7)	1107 (57.1)	12 000 (45.6)
Oxycodone	3716 (15.2)	314 (16.2)	4030 (15.3)
Morphine	488 (2.0)	19 (1.0)	507 (1.9)
Other	3534 (14.5)	124 (6.4)	3658 (13.9)

*Note* An individual can be counted in multiple groups due to the possibility of multiple dispensings on index day i.e. the sum can exceed 100%

### Postoperative one-year long-term (1yrLT) opioid use

Among all the postoperative opioid users, 3.8 were 1yrLT opioid users after surgery (i.e., at day 90, 180, *and* 365) (N 26 321) (Table 3). Among previous opioid users, the proportion was 13.1% (N 24 382) compared to 0.4% (N 1939) among new opioid users. In total 40.0% of the previous opioid users had only one opioid dispensing the year prior to surgery, whereof 1.3% were 1yrLT opioid users postoperatively. Furthermore, 13.0% of the previous opioid users were dispensed >10 prescriptions of opioids in the preceding year, and among these 49.0% were long-term users one-year after surgery (Additional file 3 a and b).

The proportion of 1yrLT opioid users was similar in men and women among the new users of opioids (Table 3). However, in previous opioid users, the proportion was higher in women than in men in all age groups (data not shown), and this sex difference increased with age (data not shown). Most long-term opioid users were dispensed codeine or tramadol as their first opioid in the first long-term use episode.

**Table 3:** Postoperative one-year long-term opioid use (1yrLT) among previous and new opioid users by age groups and sex

	Previous opioid users *n* = 185 968		New opioid users *n* = 507 527		Total *n* = 693 495	
	Total	1yrLT	Total	1yrLT	Total	1yrLT
Long-term users, *n* (%)	185 968	24 382 (13.1)	507 527	1939 (0.4)	693 495	26 321 (3.8)
Women	111 264	15 932 (14.3)	260 741	1035 (0.4)	372 005	16 967 (4.6)
Men	74 704	8450 (11.3)	246 786	904 (0.4)	321 490	9354 (2.9)
Age groups, *n* (%)						
15–39	41 256	2908 (7.0)	185 876	279 (0.2)	227 132	3178 (1.4)
40–59	69 801	9170 (13.1)	173 893	586 (0.3)	243 694	9756 (4.0)
60–79	58 070	8090 (13.9)	129 145	716 (0.6)	187 215	8806 (4.7)
80+	16 841	4214 (25.0)	18 613	358 (1.9)	35 454	4572 (12.9)

### Previous use of benzodiazepines and Z-drugs

The proportion of 1yrLT opioid users among previous opioid users who also had used Z-drugs alone, benzodiazepines alone, or both Z-drugs and benzodiazepines was 8.3%, 12.2%, and 18.3%, respectively. The proportion was particularly high among patients aged 80+ years that used both Z-drugs and benzodiazepines in the year preceding surgery (women 22.8%; men 22.1%).

### **Proportion of 1yrLT opioid users across surgical procedures**

Transluminal endoscopy, eye surgery and assessments related to surgical procedures had the highest proportion of 1yrLT opioid users among both previous users (transluminal endoscopy = 26.3%; eye surgery = 24.7%; assessments related to surgical procedures = 23.8%) and new users of opioids (transluminal endoscopy = 1.5%; eye surgery = 1.1%; assessments related to surgical procedures = 1.3%). The difference in the proportion of 1yrLT opioid users between previous and new users of opioids were also evident across surgical procedures (Table 4). We also found a difference in the proportion of previous opioid use depending on the type of surgery (Additional file 3). For instance, 60% of patients that received transluminal endoscopy were previous users of opioids. These patients had on mean eight and a median of five opioid dispensings the year prior to surgery.

**Table 4:** Proportion of patients with one-year long-term opioid use (1yrLT) among previous and new users of opioids according to categories of surgical procedures, i.e., NOMESCO main chapters

NCSP chapters *N* (%)	Previous opioid users		New opioid users	
	Total	1yrLT	Total	1yrLT
Nervous system	16 991	1685 (9.9)	20 971	111 (0.5)
Endocrine organs	246	43 (17.4)	599	-
Eye	6434	1589 (24.7)	5706	63 (1.1)
Ear, nose, sinus, larynx	5150	572 (11.1)	22 504	26 (0.1)
Teeth, jaw, mouth and pharynx	6657	305 (4.5)	34 709	15 (0.04)
Heart and the large internal thoracic veins	1760	341 (19.3)	3992	29 (0.7)
Chest	801	144 (17.9)	3143	31 (0.9)
Mammae	1235	93 (7.5)	4524	-
Digestive organs and spleen	19 704	2365 (12.0)	58 031	182 (0.3)
Urinary organs, male genitalia and retroperitoneal room	3789	609 (16.0)	7927	34 (0.4)
Female genitalia	6456	1007 (15.6)	14 470	28 (0.1)
Birth, pregnancy	502	76 (15.1)	1596	-
Locomotor system	66 031	5092 (7.7)	233 607	776 (0.3)
Peripheral veins and lymph system	2516	403 (16.0)	7221	54 (0.7)
Skin	11 818	1973 (16.6)	30 445	165 (0.5)
Minor surgical procedures	16 363	3048 (18.6)	45 079	221 (0.4)
Transluminal endoscopy	15 213	4013 (26.3)	10 541	167 (1.5)
Assessments related to surgical procedures	4285	1021 (23.8)	2345	32 (1.3)
Extraction of organ or tissue for transplantation	17	-	117	-

*Note* “Proportions” representing *n* < 5 for the different NCSP main chapters are not displayed

### Proportion of one-year long-term opioid users according to health region

We found differences in the proportion of 1yrLT postoperative opioid users across the health regions in Norway, with the highest proportion in the Northern (4.3%) region followed by the South-Eastern (3.9%), Western (3.6%) and the Central (3.3%) regions.

## Discussion

This study included the postoperative population among individuals aged ≥ 15 that were dispensed opioids in Norway and we found that the proportion of one-year long-term opioid use, with at least 10 MME/day in the initial 90 days, is low. Previous opioid users were typically older and female, compared to new users.

The proportion of 1yrLT in new opioid users was lower than previously reported. A recent study from Sweden reported a prevalence of 7% among opioid naïve patients that had undergone major surgery [18]. A similar population-based Danish study included elderly (≥65) opioid-naïve hip fracture surgery patients, and found a prevalence of long-term opioid use of 15% [19]. One Canadian study included opioid-naïve elderly patients undergoing low-risk surgery, and found a prevalence of long-term opioid use of 8% among the surgery patients [20]. Brummet and colleagues found that in new users of opioids, 6% were long-term opioid users after surgery [4]. Others have reported a prevalence of long-term opioid use in opioid naive patients of 8% [21]. Common to these studies is the use of the surgical population as the denominator, while our proportions refer to the postoperative opioid users as the denominator. Still, our proportion measures were much lower than in these studies. We cannot rule out that differences in study country, included surgery types, and definitions of opioid use may have contributed to the discrepancy between our study and previous comparable studies. The prevalence measures of persistent analgesic use can vary several folds depending on the definition [16, 22, 23]. A direct comparison of our proportion measures with other studies, conducted with other definitions and in other countries, is therefore challenging. Thus, we consider the internal differences, i.e., differences between sub groups, in our study to be more of an interest.

Our results support the repeatedly reported relation between previous exposure to opioids and prolonged opioid use after surgery [24, 25]. In our study, 0.4% among the new users of opioids were one-year long-term users after surgery, compared to 13.0% among the previous opioid users. Among the 13.0% of the previous opioid users that were dispensed >10 prescriptions of opioids, 49.0% were long-term users one-year after surgery. Thus, previous opioid use seems to increase the likelihood of long-term use. This may be due to conditions other than the surgery. It should be highlighted that 99.6% of the new and 87% of the previous opioid users did not have persistent opioid use after surgery, according to our definition, possibly implying successful pain management, weaning and positive surgical outcomes for the majority of the patients. However, the group of previous opioid users was heterogenous, with varying numbers of opioid dispensings the year prior to surgery and differing proportion measures of previous users across the surgery groups.

Recent reviews have compiled the existing literature [26, 27]. While these articles may incorporate studies that did not fully comprise with the methodology employed in the present study, the prevalence figures they provide are noteworthy and may highlight important differences in opioid utilization and prescription policies, and their consequences across countries. One systematic review evaluated data on long-term postoperative opioid use from 33 studies and reported a pooled prevalence rate of long-term opioid use of 6.7% in all surgery patients and 1.2% in opioid-naïve patients [25]. Despite methodological differences, the findings are somewhat corresponding to our study.

Even though the proportion of one-year long-term opioid use was low in the population as a whole and in most age groups, it increased with age, and displayed quite high proportions among those who had used opioids previous to surgery in the oldest age group, 80 years old and older (25%). As previously reported [17], we found that more females than males were long-term users of opioids after surgery. Except for the youngest age group, aged 15–39 years old, more females than males were dispensed opioids after surgery. Additionally, females surpassed males in long-term opioid use post-surgery across all the age groups, and this difference increased with age. Thus, more females received opioids and had long-term opioid use after surgery, and the sex difference in long-term opioid use was most pronounced in the oldest patient group (>79 years old). Sex differences in chronic pain and opioid use is well known in the scientific literature, with females being more prone to chronic pain and higher use of opioids [28]. Our study did not apply a methodological approach enabling further explanation of the sex differences, but previous studies have highlighted comorbidity and the interplay between biological and psychological factors [29], which are likely to be relevant here as well.

Patients undergoing eye surgery, transluminal endoscopy and assessments related to surgical procedures had the highest proportion of one-year long-term opioid use. The existing knowledge about prevalence of long-term opioid use after ophthalmic surgery is sparse. Ung and colleagues examined long-term opioid use in opioid-naïve eye surgery patients that received a perioperative opioid prescription during the 30 days prior to surgery [30]. They reported that long-term opioid use was found in in 3.4% of the patients, which is higher than in our study (1.1%). New, advanced surgical techniques within cataract surgery leads to more extensive use of topical or incremental anesthesia, and less use of opioids peri- and postoperatively. Within other surgical disciplines, it has been shown that a switch to new, less invasive and robotic-assisted surgery reduce opioid prescribing and long-term use [31]. In our study, cataract surgery was by far the most frequent surgical procedure among eye patients (Additional file 4). As cataract surgery is mostly performed in elderly patients [32], our finding of high proportion of long-term opioid use in this patient group may be an age effect, e.g., related to comorbidity.

Transluminal endoscopic surgery and assessments related to surgical procedures is often performed in the diagnostic assessment of several serious diseases [33]. Although we did exclude patients with cancer the year previous to surgery, we cannot rule out the possibility that the high proportion of one-year long-term opioid use in patients undergoing transluminal endoscopy is due to cancer or the detection of other serious diseases, and thus opioids being part of their treatment for their background illness in the year following surgery. Gastroscopy, rhinopharyngoscopy, enteroscopy and coloscopy constituted 73% of the transluminal endoscopic procedures in our study. As several of these procedures are commonly performed in the examination of the presence or severity of tumors or cancer, our findings may reflect opioid use related to these conditions and not the surgery.

### Strengths and limitations

This study captured the whole population of surgery patients that filled an opioid prescription after surgery in Norway over an 8 year span. A significant advantage is its comprehensive overview of opioid use in the surgery population, a perspective that has not previously been explored in a Nordic setting. The inclusion of national, reliable and complete data enhances the study´s robustness and generalizability. This overview provides a broad perspective on postoperative opioid use, and sheds light on surgery related opioid prescription practices, opioid use and health challenges in Norway.

The registers do not hold data on in-hospital opioid use, which in Denmark has been found to constitute approximately 4% of the total opioid consumption volume [34]. Furthermore, even though our definition of long-term opioid use required more than one dispensing and may indicate that the medications were used, our data reflects opioid dispensing, not necessarily actual use. We excluded opioids used in opioid maintenance treatment, as these drugs are mostly not clinically relevant for the surgical population. However, they may be used for pain management in a few situations. Furthermore, the lack of consensus of definitions of long-term opioid use and opioid naïvety in the literature represents a challenge when comparing our results with results from other studies.

The data on surgical procedures were grouped according to NOMESCO and limited to the third digit due to data protection restrictions. The NOMESCO coding system was extensively revised and adjusted during the study period. Due to the resolution of our data and our definition of surgery, we were unable to separate for instance diagnostic procedures from larger surgeries. The revision of the NCSP codes, with certain surgical procedures moved between NCSP main chapters, may also have affected the proportion measures in the different categories. Finally, we selected patients that were required to survive for at least 365 days after the index date. Our study cohort therefore represents a “healthier cohort” and our study findings are mostly applicable to patients with a longer life expantancy after surgery. Thus, the findings from the present study should be interpreted cautiously. Despite these limitations, the findings could provide guidance for future studies and identify possible areas for further examination and intervention.

## Conclusions

One-year long-term postoperative opioid use among all postoperative opioid users in Norway is low, but previous exposure to opioids may increase the likelihood of long-term postoperative use. Long-term opioid use is more frequent in females than in males, and increase with age. Vigilance towards postoperative opioid prescribing, particularly among previous opioid users, is warranted to prevent progression to long-term use.

## Electronic supplementary material

Below is the link to the electronic supplementary material.

Supplementary Material 1 Additional file 1. The NOMESCO Classification of Surgical Procedures. NOMESCO, The Nordic Medico-Statistical Committee, NCSP = NOMESCO Classification of Surgical Procedures

Supplementary Material 2 Additional file 2. Study diagram. Visual representation of the study setup, with illustration of the timeline for washout windows, inclusion and exclusion criteria, covariates and time period for the definition of long-term opioid use

Supplementary Material 3 Additional file 3. The relationship between number of opioid dispensings in the washout period and one-year long-term use (1yrLT). The number of previous opioid users that had exactly (circles) and at least (bullets) 1, 2,…, 51+ dispensings 1-365 days before index date. **b)** Percentage of patients that had one-year long-term use (1yrLT), among patients with exactly (circles) and at least (bullets) 0, 1, 2,… 51+ opioid dispensings the year before index date. The circle at *N* = 0 and the bullet at *N* = 1 indicate the proportion with 1yrLT in new and previous users, respectively. **c)** Percentage of previous opioid users by surgery chapter. **d)** Mean (with 95% confidence intervals) and median number of dispensings the year before index date for previous opioid users, by surgery chapter

Supplementary Material 4 Additional file 4. The 10 most frequent surgical procedures in the study population in each NOMESCO main chapter. Values < 5 are not shown. Unlabeled code descriptions are unknown and possibly reflect outdated codes that are no longer in use in the NOMESCO classification system. NCSP = NOMESCO Classification of Surgical Procedures. Note: Values < 5 are not shown. Unlabeled code descriptions are unknown and possibly reflect outdated codes that are no longer in use in the NOMESCO classification system. NCSP = NOMESCO Classification of Surgical Procedures

Supplementary Material 5 Additional file 5. Proportion of one-year long-term use (1yrLT) at days 90, 180, and 365 after surgery. Note. 90 + 180: Long-term opioid use at day 90 AND 180 but NOT 365. Any: Long-term opioid use at DAY 90, OR 180, OR 365. 1yrLT: Long-term opioid use at day 90 AND 180 AND 365

## Data Availability

No datasets were generated or analysed during the current study.

## Declarations

### Disclaimer

Data from the Norwegian Patient Registry and Cancer Registry of Norway have been used in this publication. The interpretation and reporting of these data are the sole responsibility of the authors, and no endorsement by the register holders is intended nor should be inferred.
