# Supplementary material for: Postoperative opioid use in Norway—a population-based observational study on patterns of long-term use
Source: BMC Pharmacol Toxicol. 2024 Oct 25;25:81. doi: 10.1186/s40360-024-00805-y (PMC11515196; doi:10.1186/s40360-024-00805-y)
Supplement: Supplementary file 1 — Supplementary Material 1 Additional file 1. The NOMESCO Classification of Surgical Procedures. NOMESCO, The Nordic Medico-Statistical Committee, NCSP = NOMESCO Classification of Surgical Procedures [file 40360_2024_805_MOESM1_ESM.docx]

**Additional file 1. The NOMESCO Classification of Surgical Procedures**

| **NCSP codes** | **NOMESCO chapter** |
| --- | --- |
| A | Nervous system |
| B | Endocrine organs |
| C | Eye and eye region |
| D | Ear, nose, sinus and larynx |
| E | Teeth, jaw, mouth and pharynx |
| F | Heart and the large internal thoracic veins |
| G | Chest wall, pleura, diaphragma, trachea, bronchies, lungs, mediastinum |
| H | Mammae |
| J | Digestive organs and spleen |
| K | Urinary organs, male genitalia and the retroperitoneal room |
| L | Female genitalia |
| M | Birth and pregnancy |
| N | The locomotor system |
| P | Peripheral veins and the lymph system |
| Q | Skin |
| T | Minor surgical procedures |
| U | Transluminal endoscopy |
| X | Assessments related to surgical procedures |
| Y | Extraction of organ or tissue for transplantation |

*Note:* NOMESCO = The Nordic Medico-Statistical Committee, NCSP= NOMESCO Classification of Surgical Procedures
