# Supplementary figures and images for: Postoperative opioid use in Norway—a population-based observational study on patterns of long-term use
Source: BMC Pharmacol Toxicol. 2024 Oct 25;25:81. doi: 10.1186/s40360-024-00805-y (PMC11515196; doi:10.1186/s40360-024-00805-y)

**Additional file 2. Study diagram.**

**
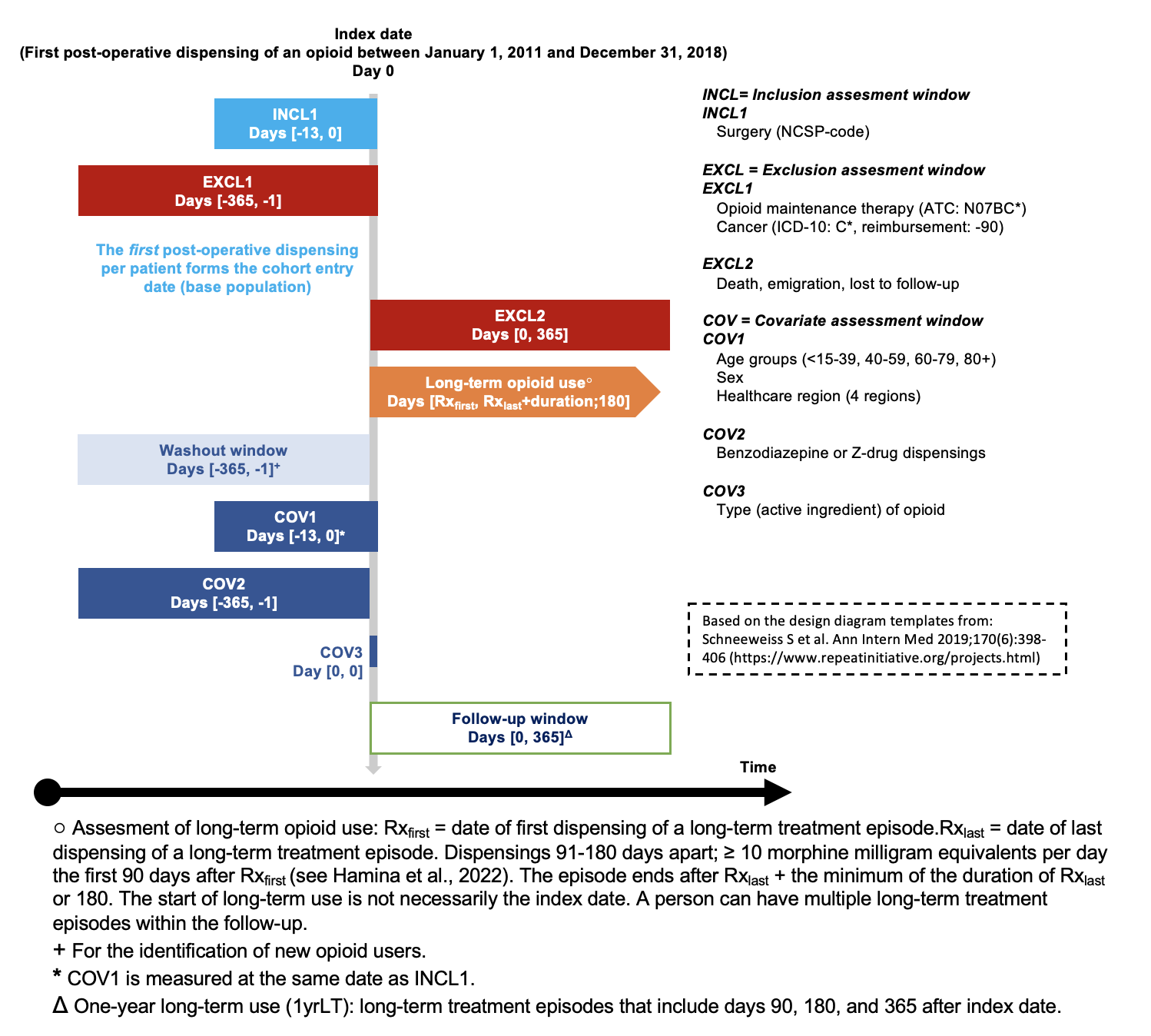
**

Supplement: Supplementary file 2 — Supplementary Material 2 Additional file 2. Study diagram. Visual representation of the study setup, with illustration of the timeline for washout windows, inclusion and exclusion criteria, covariates and time period for the definition of long-term opioid use [file 40360_2024_805_MOESM2_ESM.docx]
