# Supplementary material for: Postoperative opioid use in Norway—a population-based observational study on patterns of long-term use
Source: BMC Pharmacol Toxicol. 2024 Oct 25;25:81. doi: 10.1186/s40360-024-00805-y (PMC11515196; doi:10.1186/s40360-024-00805-y)
Supplement: Supplementary file 3 — Supplementary Material 3 Additional file 3. The relationship between number of opioid dispensings in the washout period and one-year long-term use (1yrLT). The number of previous opioid users that had exactly (circles) and at least (bullets) 1, 2,…, 51+ dispensings 1-365 days before index date. b) Percentage of patients that had one-year long-term use (1yrLT), among patients with exactly (circles) and at least (bullets) 0, 1, 2,… 51+ opioid dispensings the year before index date. The circle at N = 0 and the bullet at N = 1 indicate the proportion with 1yrLT in new and previous users, respectively. c) Percentage of previous opioid users by surgery chapter. d) Mean (with 95% confidence intervals) and median number of dispensings the year before index date for previous opioid users, by surgery chapter [file 40360_2024_805_MOESM3_ESM.docx]

**Additional file 3. The relationship between number of opioid dispensings in the washout period and one-year long-term use (1yrLT)**

**a)** The number of previous opioid users that had exactly (circles) and at least (bullets) 1, 2, … , 51+ dispensings 1-365 days before index date. **b)** Percentage of patients that had one-year long-term use (1yrLT), among patients with exactly (circles) and at least (bullets) 0, 1, 2, … 51+ opioid dispensings the year before index date. The circle at N=0 and the bullet at N=1 indicate the proportion with 1yrLT in new and previous users, respectively. **c)** Percentage of previous opioid users by surgery chapter. **d)** Mean (with 95% confidence intervals) and median number of dispensings the year before index date for previous opioid users, by surgery chapter.
