# Supplementary material for: Postoperative opioid use in Norway—a population-based observational study on patterns of long-term use
Source: BMC Pharmacol Toxicol. 2024 Oct 25;25:81. doi: 10.1186/s40360-024-00805-y (PMC11515196; doi:10.1186/s40360-024-00805-y)
Supplement: Supplementary file 4 — Supplementary Material 4 Additional file 4. The 10 most frequent surgical procedures in the study population in each NOMESCO main chapter. Values < 5 are not shown. Unlabeled code descriptions are unknown and possibly reflect outdated codes that are no longer in use in the NOMESCO classification system. NCSP = NOMESCO Classification of Surgical Procedures. Note: Values < 5 are not shown. Unlabeled code descriptions are unknown and possibly reflect outdated codes that are no longer in use in the NOMESCO classification system. NCSP = NOMESCO Classification of Surgical Procedures [file 40360_2024_805_MOESM4_ESM.docx]

**Additional file 4. The 10 most frequent surgical procedures in the study population in each NOMESCO main chapter.**

| **NCSP code** | **Code description** | | | | **N** | |  |
| --- | --- | --- | --- | --- | --- | --- | --- |
| ***Nervous system*** | | | | | | |  |
| ABC | Decompression of spinal cord and nerve roots | | | 21 049 | | |  |
| ACC | Surgery of dysfunction in peripheral nerve roots | | | 11 395 | | |  |
| ACB | Surgery of lesions in peripheral roots | | | 2 627 | | |  |
| AAF | Shunt surgery on brain ventricles and intracranial cysts | | | 320 | | |  |
| ACA | Diagnostisc surgery of peripheral nerves | | | 298 | | |  |
| AAC | Surgery intracranial aneurysms / other vascular lesions | | | 277 | | |  |
| ABD | Surgery on spinal cord and nerve roots for pain and functional disorders | | | 253 | | |  |
| AAK | Surgery on cranium and dura mater | | | 236 | | |  |
| AAD | Surgery for head injuries | | | 209 | | |  |
| ABB | Surgery for lesions in the spinal canal | | | 209 | | |  |
| ***Endocrine organs*** | | | | | | |  |
| BAA | Thyroid gland | | | 517 | | |  |
| BCA | Adrenal gland | | | 175 | | |  |
| BBA | Parathyroid gland | | | 95 | | |  |
| BAX | Minor procedures on thyroid gland | | | 50 | | |  |
| BDA | Glomus caroticum | | | 6 | | |  |
| BAB |  | | | <5 | | |  |
| BWE | Reoperation deep haemorrhage in endocrine surgery | | | <5 | | |  |
| Unknown |  | | | <5 | | |  |
| Unknown |  | | | <5 | | |  |
| Unknown |  | | | <5 | | |  |
|  |  | | |  | | |  |
| ***Eye, eye region*** | | | | | | |  |
| CJE | Extracapsular cataract surgery with emulsification | | | 2 512 | | |  |
| CKD | Intraocular surgery on vitreous body and retina | | | 2 132 | | |  |
| CBB | Procedures for localized changes in eyelids | | | 1 047 | | |  |
| CGG | Reconstructive procedures on cornea and sclera | | | 991 | | |  |
| CXX | Other eye examinations | | | 933 | | |  |
| CJB | Surgery for secondary cataract | | | 819 | | |  |
| CGE | Surgery for lesions in cornea and sclera | | | 591 | | |  |
| CKC | Extraocular surgery on vitreous body and retina | | | 492 | | |  |
| CHD | Filtration surgery | | | 273 | | |  |
| CAC | Surgery on the orbital bone walls | | | 271 | | |  |
| ***Ear, nose, sinus and larynx*** | | | | | | |  |
| DJD | Suture nasal septum | | | 8 435 | | |  |
| DHB | Exision outer nasal lesion | | | 6 820 | | |  |
| DUH | Rhinopharyngoscopy | | | 2 805 | | |  |
| DMB | Trepanation av maxillary sinus | | | 2 055 | | |  |
| DNB | External ethmoidectomy | | | 1 844 | | |  |
| DUQ | Transluminal endoscopy ear, nose, larynx | | | 1 150 | | |  |
| DLD | Plastic surgery nose | | | 1 093 | | |  |
| DKW | Procedures for nosebleeds | | | 550 | | |  |
| DCD | Myringoplasty or tympanoplasty | | | 417 | | |  |
| DHD | Internal suture of nose, closed or open reduction of fracture of nose, osteosynthesis of fracture nose | | | 319 | | |  |
| ***Teeth, jaw, mouth, pharynx*** | | | | | | |  |
| EMB | Resection procedures on tonsils and adenoid tissue | | | 24 272 | | |  |
| EBA | Extraction and resection of teeth | | | 6 219 | | |  |
| EDC | Reconstruction procedures on lower jaw | | | 1 696 | | |  |
| ENC | Reconstruction procedures on pharynx and adjacent structures | | | 1 357 | | |  |
| EEC | Reconstruction procedures on upper jaw | | | 1 036 | | |  |
| EBB | Reconstruction procedures on teeth | | | 957 | | |  |
| ENA | Incision and biopsy of the pharynx and adjacent structures | | | 825 | | |  |
| ELB | Resection procedures on salivary glands | | | 777 | | |  |
| EFA | Resection procedures on jaws | | | 502 | | |  |
| EMA | Incision and biopsy of tonsils and adenoid tissue | | | 347 | | |  |
| ***Heart*** | | | | | | |  |
| FNA | Anastomosis between internal mammary artery and coronary artery | | | 1 387 | | |  |
| FNG | Dilation and recanalization of coronary arteries | | | 1 065 | | |  |
| FXA | Perioperative total cardiopulmonary bypass | | | 722 | | |  |
| FPE | Replacement of transvenous pacemaker pulse generator | | | 483 | | |  |
| FMD | Implantation of aortic valve prosthesis | | | 466 | | |  |
| FPG | Implantation of permanent cardioverter-defibrillator | | | 277 | | |  |
| FPB | Excision or destruction of aberrant conduction pathway or cardiac focus | | | 174 | | |  |
| FCA | Reconstructive surgery aorta ascendens | | | 154 | | |  |
| FNC | Aortocoronary vein bypass | | | 119 | | |  |
| FPK | Implantation of heart monitoring equipment | | | 103 | | |  |
| ***Chest*** | | | | | | |  |
| GAA | Drainage and endoscopy of pleura | | | 2 010 | | |  |
| GAF | Surgery for chest wall deformities | | | 501 | | |  |
| GAE | Incision, biopsy, resection, and reconstruction of chest wall | | | 258 | | |  |
| GUC | Bronchoscopy | | | 219 | | |  |
| GDB | Minor resection procedures on lung | | | 205 | | |  |
| GAC | Surgery on pleura | | | 174 | | |  |
| GAX | Minor thoracic surgical procedures | | | 148 | | |  |
| GAB | Thoracotomy | | | 146 | | |  |
| GDA | Incision, excision of a lesion, and lung reconstruction | | | 72 | | |  |
| GEC | Syrgery on thymus | | | 58 | | |  |
| ***Mammae*** | | | | | | |  |
| HAD | Plastic surgery of mammae | | | 3 638 | | |  |
| HAB | Resection of mammae | | | 823 | | |  |
| HAC | Mastectomy | | | 728 | | |  |
| HAE | Reconstruction of mammae | | | 426 | | |  |
| HAA | Incisjon, sutur and biopsi of mammae | | | 92 | | |  |
| HWE | Reoperation for deep bleeding in mammae surgery | | | 18 | | |  |
| HWW | Other reoperations in mammae surgery | | | 9 | | |  |
| HAW | Other minor procedures of mammae | | | 8 | | |  |
| HAX | Minor procedures of mammae | | | <5 | | |  |
| HWC | Reoperation for deep infection in surgery of mammary gland | | | <5 | | |  |
| ***Digestive organs, spleen*** | | | | | | |  |
| JKA | Gallbladder surgery | | | 12 699 | | |  |
| JAB | Inguinal hernia surgery | | | 11 156 | | |  |
| JHB | Procedures for hemorrhoids and anal mucosal prolapse | | | 8 325 | | |  |
| JEA | Appendectomy | | | 8 307 | | |  |
| JUD | Gastroscopy | | | 4 097 | | |  |
| JHA | Local procedures on anus and perianal tissue | | | 3 787 | | |  |
| JAF | Umbilical hernia surgery | | | 3 704 | | |  |
| JAH | Abdominal cavity opening | | | 3 157 | | |  |
| JDF | Volume restricting intervention on ventricle | | | 2 829 | | |  |
| JUF | Enteroscopy and colonoscopy | | | 2 575 | | |  |
| ***Urinary organs*** | | | | | | |  |
| KGH | Reconstruction procedures on penis | | | 1 528 | | |  |
| KCX | Minor procedures on urinary bladder | | | 1 301 | | |  |
| KFD | Resection of the scrotum and scrotal organs | | | 1 243 | | |  |
| KUC | Cystoscopy | | | 1 046 | | |  |
| KBV | Different surgeries on ureter | | | 980 | | |  |
| KAE | Removal of stones from kidney and renal pelvis | | | 748 | | |  |
| KAT | Extracorporeal fragmentation of stones in renal pelvis | | | 718 | | |  |
| KBE | Surgeries for stones in ureter | | | 688 | | |  |
| KED | Resection of prostate | | | 603 | | |  |
| KFA | Exploration and incision of scrotum and scrotal organs | | | 257 | | |  |
| ***Female genitalia*** | | | | | | |  |
| LEF | Surgeries for uterine and vaginal apex prolapse | | | 2 566 | | |  |
| LCD | Excision of uterus | | | 2 561 | | |  |
| LCA | Intrauterine surgeries and biopsy of uterus with ligaments | | | 2 498 | | |  |
| LAF | Excision of ovary and fallopian tube | | | 1 778 | | |  |
| LCH | Termination of pregnancy | | | 1 607 | | |  |
| LXE | Other gynecological examinations | | | 1 110 | | |  |
| LCB | Incision, excision, and destruction of lesions in uterus | | | 896 | | |  |
| LBE | Excision of fallopian tube | | | 853 | | |  |
| LCC | Resection of uterus | | | 783 | | |  |
| LAC | Excision and destruction of lesions in ovary | | | 779 | | |  |
| ***Birth, pregnancy*** | | | | | | |  |
| MCA | Cesarean section | | | 599 | | |  |
| MBA | Removal of retained pregnancy products after childbirth and abortion | | | 448 | | |  |
| MAC | Surgical induction of labor or uterine stimulation | | | 356 | | |  |
| MBC | Reconstructive procedures for birth tears | | | 208 | | |  |
| MAX | Minor obstetric procedures | | | 185 | | |  |
| MAE | Delivery by vacuum extraction | | | 122 | | |  |
| MAF | Forceps delivery in vertex presentation | | | 76 | | |  |
| MAA | Intrauterine procedures on the pregnant uterus and fetus | | | 30 | | |  |
| MAG | Vaginal delivery in breech presentation | | | 22 | | |  |
| MAB | Different operations on pregnant uterus and fetus | | | 19 | | |  |
| ***Locomotor system*** | | | | | | |  |
| NFB | Implantation primary prosthesis in hip joint and thigh | | | 31 919 | | |  |
| NGD | Surgeries on knee meniscus | | | 24 632 | | |  |
| NGB | Implantation primary prosthesis in knee joint and shin | | | 20 131 | | |  |
| NHK | Surgeries on the bones in ankle and foot | | | 15 681 | | |  |
| NCJ | Fracture surgery in elbow and forearm | | | 15 681 | | |  |
| NBK | Surgeries on the bones in shoulder and upper arm | | | 12 746 | | |  |
| NHJ | Fracture surgery in ankle and foot | | | 12 178 | | |  |
| NDX | Minor procedures on wrist and hand | | | 10 349 | | |  |
| NBA | Exploratory procedures on shoulder and upper arm | | | 9 484 | | |  |
| NHX |  | | | 8 555 | | |  |
| ***Peripheral veins, lymph system*** | | | | | | |  |
| PHD | Resection of veins | | | 4 262 | | |  |
| PDG | Surgeries for aneurysm in infrarenal abdominal aorta and iliac arteries | | | 837 | | |  |
| PHB | Ligation of veins | | | 545 | | |  |
| PEF | Thromboendarterectomy in femoral artery and its branches | | | 389 | | |  |
| PDQ | | Insertion of stent into infrarenal abdominal aorta and iliac arteries | | | 313 | | |
|  |  | | |  | | |  |
| PHX |  | | | 288 | | |  |
| PJD | Excision and block dissection of lymph nodes | | | 281 | | |  |
| PHV | Endovenous obliteration of veins | | | 252 | | |  |
| PEP | Percutaneous angioplasty | | | 224 | | |  |
| PDH | Bypass from infrarenal abdominal aorta and iliac arteries | | | 222 | | |  |
| ***Skin*** | | | | | | |  |
| QCB | Surgical wound treatment of skin on upper extremities | | | 9 512 | | |  |
| QDB | Surgical wound treatment of skin on lower extremities | | | 6 243 | | |  |
| QDH | Surgeries on toenails | | | 4 279 | | |  |
| QBE | Surgeries for skin lesions on trunkus | | | 3 887 | | |  |
| QBJ | Plastical surgry on skin on trunkus | | | 2 843 | | |  |
| QAB | Surgical wound treatment on head and neck | | | 2 412 | | |  |
| QXB | Surgical wound treatment on an unspecified region of the skin | | | 1 812 | | |  |
| QBA | Incision and local destruction of skin lesion on trunkus | | | 1406 | | |  |
| QAE | Surgeries for skin lesions on head and neck | | | 1 385 | | |  |
| QBB | Surgical wound treatment on trunkus | | | 887 | | |  |
| ***Minor surgical procedures*** | | | | | | |  |
| TND | | | Wrist and hand | 17 429 | | |  |
| TNH | Ankle and foot | | | 12 837 | | |  |
| TNB | Shoulder and upper arm | | | 10 511 | | |  |
| TNC | Elbow and forearm | | | 4 916 | | |  |
| TNG | Knee joint and lower leg | | | 4 591 | | |  |
| TAB | Lumbar puncture | | | 2 060 | | |  |
| TKC | Needle biopsy, percutaneous puncture or catheterisation of bladder | | | 1 712 | | |  |
| TQX |  | | | 1 158 | | |  |
| TNF | Hip, joint and thigh | | | 641 | | |  |
| TLC | Insertion of intrauterine contraceptive device | | | 517 | | |  |
| ***Transluminal endoscopy*** | | | | | | |  |
|  |  | | |  | | |  |
| UJD | Gastroscopy | | | 8 561 | | |  |
| UDH | Rhinopharyngoscopy | | | 5 924 | | |  |
| UJF | Enteroscopy and coloscopy | | | 4 291 | | |  |
| UKC | Cytoscopy | | | 1 952 | | |  |
| UDQ | Laryngoscopy | | | 1 943 | | |  |
| UJG | Protoscopy | | | 1 227 | | |  |
| UJH | Anoscopy | | | 690 | | |  |
| UGC | Bronchoscopy | | | 371 | | |  |
| UJK | Endoscopic and radiological examination of bilary tract and pancreatic duct | | | 270 | | |  |
| UEN | Oropharyngoscopy and hypopharyngoscopy | | | 156 | | |  |
| ***Assessments related to surgical procedures*** | | | | | | |  |
|  |  | | |  | | |  |
| XCW | Other investigative procedures on eye | | | 3 057 | | |  |
| XLE | Coloscopy | | | 2 799 | | |  |
| XCK | Perimetry | | | 448 | | |  |
| XJD | Peroral endoscopic ultrasonography | | | 69 | | |  |
| XJH | Transanal utrasonograhpy, anorectal manometry, electromyography of anal sphincter | | | 68 | | |  |
| XJW | Other investigative procedure on digestive system | | | 56 | | |  |
| XJC | Oesophaegal manometry, PH monitoring or electromyography | | | 31 | | |  |
| XFX | Intraoperative puncture if heart or major thoracic vessel, Intraoperative puncture manometry of heart or major thoracic vessel, Intraoperative flowmetry of heart or major thoracic vessel, Epicardial ultrasonography of heart, Other investigative intraoperative puncture of heart or major thoracic vessel, Other thoracoscopic investigative intraoperative puncture of heart or major thoracic vessel | | | 26 | | |  |
| XNX |  | | | 21 | | |  |
| XCC | Investigative irrigation of lacrimal passages | | | 13 | | |  |
| ***Organ or tissue extraction for transplantation*** | | | | | | |  |
| YKA | Extraction of kidney | | | 68 | | |  |
| YNB | Extraction of bone marrow | | | 24 | | |  |
| YNA | Extraction of tissue from the locomotor system | | | 20 | | |  |
| YQA | Extraction of skin | | | 15 | | |  |
| YPA | Extraction of blood vessels | | | 7 | | |  |
| Unknown |  | | | <5 | | |  |
| Unknown |  | | | <5 | | |  |
| Unknown |  | | | <5 | | |  |
| Unknown |  | | | <5 | | |  |
| Unknown |  | | | <5 | | |  |

*Note: Values <5 are not shown. Unlabeled code descriptions are unknown and possibly reflect outdated codes that are no longer in use in the NOMESCO classification system.* NCSP= NOMESCO Classification of Surgical Procedures
