# Supplementary material for: Postoperative opioid use in Norway—a population-based observational study on patterns of long-term use
Source: BMC Pharmacol Toxicol. 2024 Oct 25;25:81. doi: 10.1186/s40360-024-00805-y (PMC11515196; doi:10.1186/s40360-024-00805-y)
Supplement: Supplementary file 5 — Supplementary Material 5 Additional file 5. Proportion of one-year long-term use (1yrLT) at days 90, 180, and 365 after surgery. Note. 90 + 180: Long-term opioid use at day 90 AND 180 but NOT 365. Any: Long-term opioid use at DAY 90, OR 180, OR 365. 1yrLT: Long-term opioid use at day 90 AND 180 AND 365 [file 40360_2024_805_MOESM5_ESM.docx]

| **Additional file 5. Proportion of one-year long-term use (1yrLT) at days 90, 180, and 365 after surgery** | | | | | | |
| --- | --- | --- | --- | --- | --- | --- |
| **NCSP code** | Total | 90 | 180 | 365 | Any | 1yrLT |
| *New users N (%)* |  |  |  |  |  |  |
| Nervous system | 20 971 | 222 (1.06) | 223 (1.06) | 204 (0.97) | 330 (1.57) | 111 (0.53) |
| Endocrine organs | 599 |  |  |  |  |  |
| Eye, eye region | 5 706 | 115 (2.02) | 119 (2.09) | 116 (2.03) | 174 (3.05) | 63 (1.01) |
| Ear, nose, sinus and larynx | 22 504 | 53 (0.24) | 53 (0.24) | 57 (0.25) | 87 (0.399 | 26 (0.12) |
| Teeth, jaw, mouth, pharynx | 34 709 | 38 (0.11) | 34 (0.10) | 48 (0.14) | 72 (0.21) | 15 (0.04) |
| Heart | 3 992 | 66 (1.65) | 72 (1.80) | 60 (1.50) | 100 (2.51) | 29 (0.73) |
| Chest | 3 143 | 70 (2.23) | 57 (1.81) | 48 (1.53) | 89 (2.83) | 31 (0.99) |
| Mammae | 4 524 |  | 8 (0.18) | 12 (0.27) | 15 (0.33) |  |
| Digestive organs, spleen | 58 031 | 356 (0.61) | 366 (0.63) | 386 (0.67) | 579 (0.67) | 182 (0.31) |
| Urinary organs | 7 927 | 76 (0.96) | 78 (0.98) | 77 (0.97) | 123 (1.55) | 34 (0.43) |
| Female genitalia | 14 470 | 43 (0.30) | 60 (0.41) | 88 (0.61) | 109 (0.75) | 28 (0.19) |
| Birth, pregnancy | 1 596 | 6 (0.38) | 5 (0.31) | 9 (0.56) | 12 (0.75) |  |
| Locomotor system | 233 607 | 2191 (0.94) | 1785 (0.76) | 1430 (0.61) | 2930 (1.25) | 776 (0.33) |
| Peripheral veins, lymph system | 7 221 | 102 (1.41) | 93 (1.29) | 103 (1.43) | 155 (2.15) | 54 (0.75) |
| Skin | 30 445 | 307 (1.01) | 314 (1.03) | 300 (0.99) | 452 (1.48) | 165 (0.54) |
| Minor surgical procedures | 45 079 | 497 (1.10) | 442 (0.98) | 425 (0.94) | 713 (1.58) | 221 (0.49) |
| Transluminal endoscopy | 10 541 | 302 (2.87) | 300 (2.85) | 300 (2.85) | 445 (4.22) | 167 (1.58) |
| Assessments related to surgical procedures | 2 345 | 49 (2.09) | 49 (2.09) | 51 (2.17) | 69 (2.94) | 32 (1.36) |
| Organ or tissue extraction for transplantation | 117 |  |  |  |  |  |
| Total | 507 527 | 4493 (0.9) | 4058 (0.8) | 3714 (0.7) | 6454 (1.3) | 1934 (0.4) |
| *Previous users N (%)* | | | | | | |
|  |  |  |  |  |  |  |
| Nervous system | 16 991 | 2090 (12.30) | 2206 (12.98) | 2279 (13.41) | 2722 (16.02) | 1685 (9.92) |
| Endocrine organs | 246 | 51 (20.73) | 55 (22.36) | 55 (22.36) | 64 (26.02) | 43 (17.48) |
| Eye, eye region | 6 434 | 1856 (28.85) | 1919 (29.83) | 1912 (29.72) | 2185 (33.96) | 1589 (24.70) |
| Ear, nose, sinus and larynx | 5 150 | 671 (13.03) | 704 (13.67) | 729 (14.16) | 831 (16.14) | 572 (11.11) |
| Teeth, jaw, mouth, pharynx | 6 657 | 348 (5.23) | 375 (5.63) | 397 (5.96) | 444 (6.67) | 305 (4.58) |
| Heart | 1 760 | 393 (22.33) | 412 (23.41) | 435 (24.729 | 487 (27.67) | 341 (19.38) |
| Chest | 801 | 179 (22.35) | 179 (22.35) | 184 (22.97) | 220 (27.47) | 144 (17.98) |
| Mammae | 1 235 | 98 (7.94) | 107 (8.66) | 114 (9.23) | 119 (9.64) | 93 (7.53) |
| Digestive organs, spleen | 19 704 | 2803 (14.23) | 2949 (14.97) | 3028 (15.37) | 3490 (17.71) | 2365 (12.00) |
| Urinary organs | 3 789 | 728 (19.21) | 770 (20.32) | 761 (20.08) | 893 (23.57) | 609 (16.07) |
| Female genitalia | 6 456 | 1139 (17.64) | 1206 (18.68) | 1271 (19.69) | 1411 (21.86) | 1007 (15.60) |
| Birth, pregnancy | 502 | 78 815.54) | 90 (17.93) | 101 (20.12) | 104 (20.72) | 76 (15.14) |
| Locomotor system | 66 031 | 6759 (10.24) | 6752 (10.23) | 6523 (9.88) | 8271 (12.53) | 5092 (7.71) |
| Peripheral veins, lymph system | 2 516 | 512 (20.35) | 514 (20.43) | 498 (19.79) | 614 (24.40) | 403 (16.02) |
| Skin | 11 818 | 2416 (20.44) | 2459 (20.81) | 2412 (20.41) | 2872 (24.30) | 1973 (16.69) |
| Minor surgical procedures | 16 363 | 3530 (21.57) | 3703 (22.63) | 3791 (23.17) | 4295 (26.25) | 3048 (18.63) |
| Transluminal endoscopy | 15 213 | 4557 (29.95) | 4832 (31.76) | 4970 (32.67) | 5550 (36.48) | 4013 (26.38) |
| Assessments related to surgical procedures  Legge inn totalrad? | 4 285 | 1140 (26.60) | 1226 (28.61) | 1273 (29.71) | 1397 (32.60) | 1021 (23.83) |
| Organ or tissue extraction for transplantation | 17 |  |  |  |  |  |
| Total | 185 968 | 29 348 (15.8) | 30 458 (16.4) | 30 733 (16.5) | 35 969 (19.3) | 24 379 (13.1) |

*Note. Any: Long-term opioid use at DAY 90, OR 180, OR 365. 1yrLT: Long-term opioid use at day 90 AND 180 AND 365.*
